# 2′-Fucosyllactose supplementation results in a transient improvement in gut microbial resilience after vancomycin use in adults with overweight or obesity: a randomized, double-blind, placebo-controlled intervention

**DOI:** 10.1080/19490976.2025.2580693

**Published:** 2025-11-16

**Authors:** Lars M.M. Vliex, David Barnett, Elena Monzel, Arjen Nauta, Guy J. Oudhuis, Marina Fassarella, Jens J. Holst, Erwin G. Zoetendal, John Penders, Ellen E. Blaak

**Affiliations:** a Department of Human Biology, NUTRIM Institute of Nutrition and Translational Research in Metabolism, Maastricht University Medical Center+, Maastricht, The Netherlands; b Department of Medical Microbiology, Infectious Diseases and Infection Prevention, NUTRIM Institute of Nutrition and Translational Research in Metabolism, Maastricht University Medical Center+, Maastricht, The Netherlands; c FrieslandCampina, Amersfoort, The Netherlands; d Laboratory of Microbiology, Wageningen University & Research, Wageningen, The Netherlands; e The NovoNordisk Foundation Center for Basic Metabolic Research and Department of Biomedical Sciences, Faculty of Health and Medical Sciences, University of Copenhagen, Copenhagen, Denmark

**Keywords:** Gut microbiome, antibiotics, prebiotics, metabolic health

## Abstract

Antibiotic-induced perturbations of the gut microbiome can be long-lasting and potentially affect host metabolic health. Strategies supporting microbial resilience are needed to mitigate the negative impact of antibiotics. We investigated the potential of 2′-fucosyllactose (2′-FL) supplementation after vancomycin use in a double-blind placebo-controlled randomized intervention among adults with overweight/obesity. Participants received oral vancomycin for seven days followed by 2′-FL or placebo for eight weeks. At baseline, after vancomycin use and after supplementation, glucose tolerance, insulin sensitivity, plasma lipids, glucagon-like peptide 1, inflammatory cytokines, fecal short-chain fatty acids (SCFAs) and branched-chain fatty acids were analyzed. Gut microbial diversity, composition and resilience were analyzed using 16S rRNA gene sequencing. Vancomycin use decreased gut microbial richness and diversity and disrupted microbiota composition and fecal SCFA concentrations. 2′-FL improved gut microbial resilience compared to placebo (*p*Treatment*Time = 0.043) after two weeks of supplementation, but differences were no longer observed at the end of the intervention. Two-week 2′-FL supplementation also differentially impacted specific bacterial taxa. Eight-week 2′-FL supplementation decreased fasting plasma interleukin−6 (IL−6) concentrations (*p*Treatment*Time = 0.041). 2′-FL intake led to transient improvements in gut microbial resilience after vancomycin use, indicating its beneficial potential to limit antibiotic-induced perturbations. Subsequent effects on metabolic health were limited and require further study.

## Introduction

The human gut microbiome plays an essential role in many processes in the human body, including maintenance of body weight and regulation of metabolic health.^1^^,^^2^ The impact of these processes on host health is dependent on gut microbial composition and function, which are mainly determined by host factors as well as diet, physical activity, medication, and other lifestyle factors.^3^^,^^4^ Differences in microbial composition, diversity and function have been shown between healthy, lean individuals and individuals with obesity, as well as individuals with metabolic diseases such as type 2 diabetes mellitus (T2DM).^5-7^ Furthermore, specific gut microbial patterns have been associated with tissue-specific insulin sensitivity, underlining the connection between the gut microbiome and host metabolic health.^8^

While generally stable, the gut microbiome can be perturbed by strong pulses such as antibiotic therapy and major alterations in diet, and these perturbations may consequently affect host health.^9^ Antibiotic therapy, often successfully used to treat bacterial infections, has been shown to clearly disrupt gut microbial composition and function.^10^^,^^11^ The antibiotic vancomycin is poorly absorbed when taken orally, meaning it reaches the colon in high doses where it affects gut microbial composition and function.^12^^,^^13^ Multiple human intervention studies have investigated the impact of oral vancomycin use on the gut microbiome, and these consistently show disruptions of microbial richness, diversity, and composition.^14-16^ While the disruptive impact of oral vancomycin use on the gut microbiome is clear, subsequent changes in host metabolic health are less evident. One study reports decreased peripheral insulin sensitivity after seven-day oral vancomycin use in males with obesity and metabolic syndrome.^14^ Conversely, two other studies report no changes in (tissue-specific) insulin sensitivity after either seven-day or 14-d oral vancomycin use (different doses) compared to control in males with obesity and insulin resistance and adults with obesity and impaired glucose tolerance, respectively.^15^^,^^16^

Remarkably, the gut microbiome is resilient and has the capacity to recover to its initial state after perturbation, although the degree of recovery and the speed at which this occurs differs per individual.^17^ One major determinant of gut microbial resilience is the composition and function of the baseline microbiome, which, as described above, is shaped by intrinsic and extrinsic influences.^18^ Consequently, microbial resilience may hold an important role in host health and disease, as it determines how the microbiota re-establishes its composition and function after disturbance, thereby shaping host physiology and immune response.^9^ In the context of antibiotic-induced disruptions, stimulating microbial resilience and thus improving the speed of microbial recovery would be important to limit long-term disruption of the gut ecosystem after antibiotic use, as well as potentially improve host insulin sensitivity and metabolic health. Supplementation with prebiotics, substrates that are selectively utilized by host microorganisms conferring health benefits, may be a promising strategy to strengthen the gut microbiome and support resilience.^19^

Human milk oligosaccharides (HMOs), naturally present in breastmilk, are a set of molecules that have been shown to have prebiotic properties. 2′-fucosyllactose (2′-FL) is the most abundant HMO in human breastmilk, and previous studies have shown its ability to modulate the gut microbiome of infants by promoting growth of *Bifidobacterium spp*., as well as to confer further benefits to the host such as immune modulation and protection against pathogens.^20-22^ Additionally, consumption of 10 g/d 2′-FL for two weeks increased relative abundance of bifidobacteria in healthy adults.^23^ Bifidobacteria may improve host health by producing lactate and short-chain fatty acids (SCFAs) which can directly impact the host, but also stimulate the growth of other beneficial bacteria such as butyrate-producers through microbial cross-feeding.^24^^,^^25^ These microbial metabolites are of importance in regulating host metabolic health through their actions in peripheral tissues such as skeletal muscle, adipose tissue and liver.^26^ Finally, a recent study showed that acute 2′-FL intake (12 g) increased postprandial plasma butyrate concentration in males with obesity, as well as acetate and butyrate concentrations in lean males, which may in the long-term translate to a beneficial effect of 2′-FL supplementation for host metabolic health.^27^

Although beneficial effects of 2′-FL supplementation on gut microbial composition have been shown and putative effects on host health have been suggested, its impact after antibiotic use is unstudied. Here, we aimed to investigate the potential of 2′-FL supplementation to improve gut microbial resilience and host metabolic health after oral vancomycin use in adults with overweight or obesity. To this end, a double-blind placebo-controlled randomized human intervention study was performed.

## Methods

### Study design and inclusion criteria

Thirty-seven adults (20−65 y) with overweight or obesity (body mass index (BMI): 25–40 kg/m^2^ and no impaired fasting glucose (fasting glucose < 6.1 mmol/L) or impaired glucose tolerance (2-h glucose < 7.8 mmol/L) were included in this study (www.Clinicaltrials.gov, NCT04561284). Participants were weight stable (change in body weight <3 kg) for at least three months before starting the study. Volunteers were excluded if they were diagnosed with T2DM, prediabetes, cardiovascular disease, kidney disease, cancer, asthma or bronchitis, liver malfunction, diseases affecting glucose tolerance, gastrointestinal disease or any other major illness with a life expectancy <5 y. Other exclusion criteria included use of antibiotics in the past three months, known allergic reactions to antibiotic use, abdominal surgery, use of laxatives, regular use of prebiotic or probiotic supplementation or use <3 months prior to the start of the study, excessive use of alcohol, drugs or excessive smoking, plans to lose weight or currently following a hypocaloric diet, following a vegan diet, high-intensity exercise >3 h per week, currently pregnant, planning to become pregnant or currently breastfeeding, and use of *β*-blockers, glucose-lowering or lipid-lowering agents, statins or chronic corticosteroid treatment. Participants were recruited via advertisements in local newspapers and websites and all lived in the catchment area around Maastricht, The Netherlands. All volunteers gave written informed consent for participation in this study. The protocol was reviewed and approved by the Medical Ethical Committee of Maastricht University Medical Center + . All procedures were performed according to the Declaration of Helsinki (October 2013).

**Figure 1.**
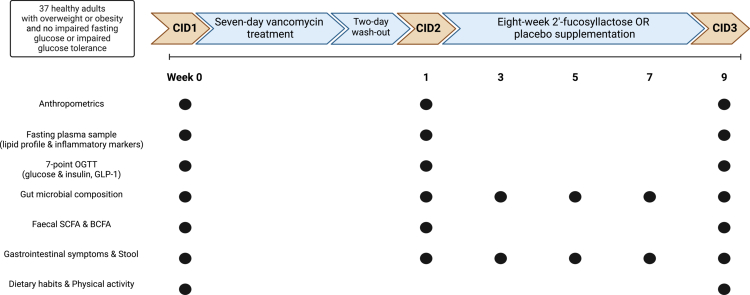
Study overview. All participants received a seven-day vancomycin treatment. After minimization, half of the participants received eight-week 2′-fucosyllactose (2′-FL) supplementation, and the other half received eight-week placebo supplementation. CID: Clinical investigation day; OGTT: oral glucose tolerance test; GLP-1: glucagon-like peptide 1; SCFA: short-chain fatty acid; BCFA: branched-chain fatty acid. Created with BioRender.com.

All volunteers enrolled in this study received oral vancomycin (3 × 500 mg/d) for seven days (Figure 1). After a two-day wash-out, participants were randomized by minimization to eight-week oral intake of either Biotis® 2′-FL (FrieslandCampina) (3 × 4 g/d) or placebo (Maltodextrin Glucidex IT 12 (Roquette)) (3 × 2 g/d). Minimisation was done using age, sex and BMI as factors. Compliance was assessed by counting returned capsules and empty sachets. Participants and investigators were blinded to the content of the sachets (2′-FL/placebo). Participants were asked to maintain their habitual diet and physical activity pattern throughout the entire study. Clinical investigation days (CID) took place at baseline (CID1), after antibiotic use and wash-out (CID2), and after supplementation (CID3). Participants were asked to consume a standard meal the evening before the CIDs. Measurements were performed on CIDs following a ten-hour overnight fast. Individuals travelled to the university by car or bus on the CIDs. Supplementary Visits (SuVs) took place after two, four and six weeks of supplementation.

### Measurements performed during Clinical investigation days and Supplementary visits

On the CIDs, body weight and waist and hip circumference were measured. During these participants took off shoes as well as heavy clothing. Blood pressure and heart rate were measured using an automatic blood pressure device (OMRON M6 Comfort). Participants underwent a 7-point oral glucose tolerance test (OGTT). For this, a cannula was inserted into the antecubital vein. Participants remained in a resting supine position throughout the test. After a fasting blood sample was drawn, participants consumed a 200 ml standard glucose drink (75 g glucose) (LemonGluc75, Novolab). Blood samples were subsequently taken at 15, 30, 45, 60, 90 and 120 minutes after consuming the drink to measure plasma glucose and insulin concentrations, as well as fasting plasma lipid profile and inflammatory markers. Plasma glucagon-like peptide 1 (GLP−1) was measured at five timepoints throughout the OGTT (t = 0, 30, 60, 90 and 120).

On both the CIDs and SuVs, participants handed in a fecal sample, which they collected the preceding day. Participants were instructed to store samples in their home freezer (-18 °C) and to transport them to the university with ice packs. The samples were then stored at -80 °C until further analysis. Finally, on the CIDs and the SuVs, participants handed in questionnaires to assess gastrointestinal complaints using the Gastrointestinal Symptom Rating Scale (GSRS) and stool consistency using the Bristol Stool Chart (BSC). At CID1 and CID3, participants handed in a three-day food record and a questionnaire on physical activity (short questionnaire to assess health-enhancing physical activity (SQUASH)).

### Biochemical analyses of plasma samples and faecal samples

Blood samples were collected in prechilled tubes and centrifuged at 3000 rpm. Plasma was snap-frozen and stored at −80 °C until analysis. Plasma glucose and free fatty acids (FFA) were measured on a Cobas Pentra C400 using ABX Pentra Glucose HK CP reagens (Horiba ABX Diagnostics, Montpellier, France) and NEFA HR reagens (Wako chemicals, Neuss, Germany), respectively. Plasma insulin was measured using ELISA (Meso Scale Discovery, Gaithersburg, USA). Plasma triglycerides, total cholesterol and high-density lipoprotein (HDL) were measured using Cobas Pentra C400 with TRIGL, CHOL2 and HDLC4 Cobas Kits (Roche Diagnostics, Rotkreuz, Switzerland). Plasma GLP−1 was measured using a radioimmunoassay with antiserum against all GLP−1 sequences, as described before.^28^ Plasma inflammatory cytokines were measured using multiplex ELISA (V-PLEX, Meso Scale Discovery, Gaithersburg, USA). Fecal short-chain fatty acid concentrations were measured using ion exchange chromatography with conductivity detection (Brightlabs, Venlo, The Netherlands) as described elsewhere.^29^ Indices of glucose metabolism and insulin sensitivity were calculated using plasma glucose, insulin and FFA concentrations (for formulas, see Supplementary methods).^30^ Low-density lipoprotein was calculated using the Friedewald formula ($\mathrm{LDL}=\;\mathrm{Total cholesterol}-\left(\frac{\mathrm{Triglycerides}}{2.2}\right)-\mathrm{HDL}$) (all in mmol/L).^31^

### Characterisation of gut microbial composition

Gut microbial composition was analyzed via the sequencing barcoded 16S ribosomal RNA (rRNA) gene amplicons using Illumina HiSeq 2500 (GATC Biotech AG for 2X150 nt), as described previously.^32^^,^^33^ In brief, DNA isolation was done by repeated bead beating of 0.25 g feces, followed by purification (automated) with a Maxwell® Tissue LEV Total RNA Purification Kit (Promega, Madison, WI). Amplification of the 16S rRNA gene V4 region was done in triplo using the double-barcoded primer pair 515F (5′-GTGCCAGCMGCCGCGGTAA)–806 R (5′-GGACTACHVGGGTWTCTAAT)^34^^,^^35^ and total bacterial DNA as a template, as described previously.^36^ An equimolar mixture of purified PCR products was used for sequencing. Raw sequence data were processed using NG-Tax 2.0 with default settings.^37^ Paired-end libraries were demultiplexed using read pairs with perfectly matching barcodes. Amplicon sequence variants (ASVs) were inferred using NG-Tax 2 denoising algorithm with default settings, and taxonomy was assigned using the SILVA database version 138.^38^ A more detailed description of this characterization can be found in the Supplementary Methods.

### Sample size calculation

A sample size of 36 was determined from an expected 12.9% difference in *Bifidobacterium* relative abundance (23) between the 2′-FL and placebo groups, with a power of 80% and a two-sided alpha of 5%. To account for a drop-out rate of 10%, 40 participants were included in the study.

### Statistical analyses

Statistical analyses were performed using R (version 4.3.2). Data are expressed as the mean + /- standard error of the mean (SEM) where applicable. The normality of metabolic parameters was inspected visually using histograms and Q-Q plots. Skewed metabolic parameters were natural log-transformed (ln) to improve normality. Differences between treatment groups at baseline were assessed using independent t tests, and differences between baseline and post-vancomycin use were assessed using paired t tests. Linear mixed effect models were used to assess changes in metabolic parameters during eight-week 2′-FL supplementation compared to placebo, with treatment and time as fixed effects and participant IDs as random effects, using the lme4 and lmerTest packages.^39^^,^^40^ The changes in metabolic parameters of interest during vancomycin use and BMI at baseline were included as covariates, unless stated otherwise. A *p*-value below 0.05 was considered significant. In the case of a significant treatment*time interaction effect, post hoc testing was done to determine differences between 2′-FL and placebo.

The microViz package (version 0.11.0—Copyright © 2022 David Barnett) was used for analysis of the gut microbial data.^41^ Unfiltered data were used for analysis of microbial richness (observed genus richness) and diversity (Shannon diversity of genera), as well as for centred-log-ratio (CLR) transformation, Aitchison distance calculations and ordination plots. For statistical modeling of individual taxa, genera with a prevalence of less than 15% across all samples were excluded, and CLR-transformed genus abundances were used. Differences between baseline and post-vancomycin use were assessed using Wilcoxon signed-rank tests, and differences between 2′-FL and placebo per timepoint were assessed using Mann-Whitney U tests. The Benjamini-Hochberg correction for multiple testing was performed, and *q* < 0.05 was considered significant.

Linear mixed effect models without covariates were fit to assess changes in bacterial taxa during eight-week 2′-FL supplementation compared to placebo, with treatment and time as fixed effects and participant IDs as random effects. First, a model was fit including all four timepoints throughout the supplementation period (eight weeks). Next, a second model was fit including only the first timepoint in the supplementation period (two weeks). In the case of a significant treatment*time interaction effect, post hoc testing was done to determine differences between 2′-FL and the placebo. For these analyses, CLR-transformed abundances were used. For visualization of genus abundance, log2-transformed taxa were used.

Microbial resilience was calculated using Aitchison distance at the genus level as a distance measure, based on the formula from Liu et al.^42^ In this formula, *d*max is the maximum Aitchison distance from baseline, and *d*(t) is the distance from baseline at timepoint t. Resilience = $\frac{d\;\max\;-d(t)}{d\;\max\;+d(t)}$. Linear mixed effect models were fit to assess changes in resilience between 2′-FL and placebo during the supplementation period, as was done for bacterial taxa. Associations between resilience and microbial parameters were assessed using Pearson correlation (richness, diversity) or Spearman correlation (taxa abundance). Associations between resilience and parameters of metabolic health were assessed using Pearson correlation.

## Results

To investigate the potential of 2′-FL supplementation after vancomycin use, a double-blind placebo-controlled randomized human intervention study was performed. Thirty-seven participants with a mean age of 44 + /−2.4 y and a mean BMI of 30.4 + /−0.7 kg/m^2^ completed the study (Table 1). There were no differences at baseline (CID1) between the groups that would eventually receive placebo or 2′-FL, except for waist and hip circumferences, which were higher in the 2′-FL group. However, the waist‒hip ratio did not differ between groups.

**Table 1.** Characteristics of the study population at baseline.

	Total (*n* = 37)		Placebo (*n* = 19)	2′-FL (*n* = 18)		*p*-value
Sex (Male/Female)	M:11/F:26		M:6/F:13	M:5/F:13		
Group (Placebo/2′-FL)	PLA:19/ 2′-FL:18					
Age (y)	44 + /−2.4		45 + /−3.3	43 + /−3.4		0.796
Weight (kg)	89.8 + /−2.9		87.2 + /−3.9	92.4 + /−4.2		0.391
BMI (kg/m^2)	30.4 + /−0.7		29.5 + /−0.9	31.4 + /−1.0		0.111
Waist (cm)	98.5 + /−2.0		94.4 + /−2.4	102.5 + /−3.0		0.044
Hip (cm)	106.3 + /−1.5		103.3 + /−1.7	109.3 + /−2.4		0.037
W/H ratio	0.92 + /−0.01		0.91 + /−0.01	0.94 + /−0.01		0.270
SBP (mmHg)	121 + /−1.7		120 + /−1.6	122 + /−3.1		0.628
DBP (mmHg)	80 + /−1.3		81 + /−1.6	80 + /−2.2		0.754
HR (bpm)	71 + /−1.7		73 + /−2.7	69 + /−2.0		0.219
Fasting Glucose (mmol/L)	5.2 + /−0.1		5.1 + /−0.1	5.2 + /−0.1		0.775
2-hour Glucose (mmol/L)	5.0 + /−0.2		4.8 + /−0.2	5.2 + /−0.3		0.222
HbA1c (mmol/mol Hb)	34.9 + /−0.5		35.2 + /−0.7	34.5 + /−0.7		0.489

2′-FL: 2′-fucosyllactose; W/H ratio: waist‒to‒hip ratio; SBP: systolic blood pressure; DBP: diastolic blood pressure; HR: heart rate; HbA1c: glycated haemoglobin.

As expected, the BSC score significantly increased during seven-day vancomycin use (3.5 + /−0.2 vs. 4.4 + /−0.2; *p* < 0.001) (Suppl. Table 1A). There was no change in gastrointestinal complaints reported in the GSRS questionnaire, although stool frequency increased (1.3 + /−0.1 stools/day vs. 1.9 + /−0.2’ *p* = 0.001) (Suppl. Table 1B, C). 2′-FL supplementation (3*4 g/day) was well tolerated: participants did not report increased gastrointestinal symptoms in the GSRS throughout the eight weeks compared to placebo. The BSC score was lower in the 2′-FL group compared to placebo after four weeks of supplementation (3.8 + /−0.2 vs. 4.6 + /−0.3; *p* = 0.049).

Participants were instructed to keep their habitual diet and physical activity throughout the study period. There was no differential change in total energy intake, macronutrient intake and dietary fibre intake between the two groups over time, nor was there a differential change in physical activity level between the two groups (Suppl. Table 2).

### Vancomycin use disrupts the gut microbiome

**Figure 2.**
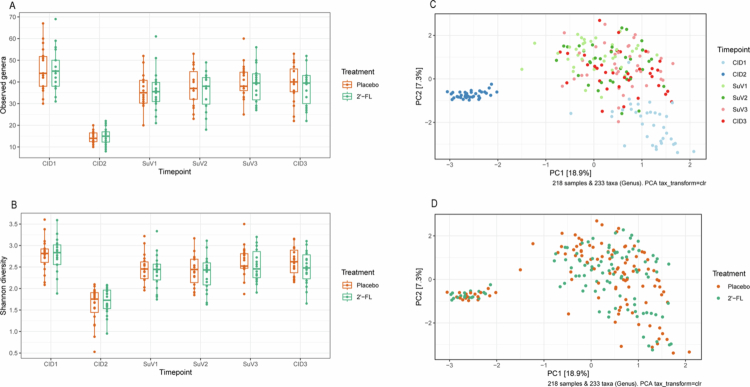
Gut microbial richness (A) and diversity (B) at baseline, after vancomycin use and throughout eight weeks of 2′-FL or placebo supplementation. Principal component analysis (PCA) based on clr-transformed genus abundance coloured by timepoint (C) or treatment (D) for the complete study period. 2′-FL: 2′-fucosyllactose, CID: Clinical Investigation Day. SuV: = Supplementary Visit. CID2, CID3, SuV1, SuV2 and SuV3 2′-FL: *n* = 18; CID1 2′-FL: *n* = 17; CID2, CID3, and SuV3 Placebo: *n* = 19; CID1, SuV1 and SuV2 Placebo: *n* = 18.

As compared to baseline, gut microbial richness and diversity were significantly depleted upon seven-day vancomycin use (observed genera: 46.0 + /−1.7 vs. 14.8 + /−0.6; Shannon diversity: 2.8 + /−0.1 vs. 1.7 + /−0.1; both *p* < 0.001) (Figure 2A-B). Furthermore, a clear shift in the gut community structure was seen after vancomycin use, as these samples clustered together and away from the samples from all other timepoints (Figure 2C).

**Figure 3.**
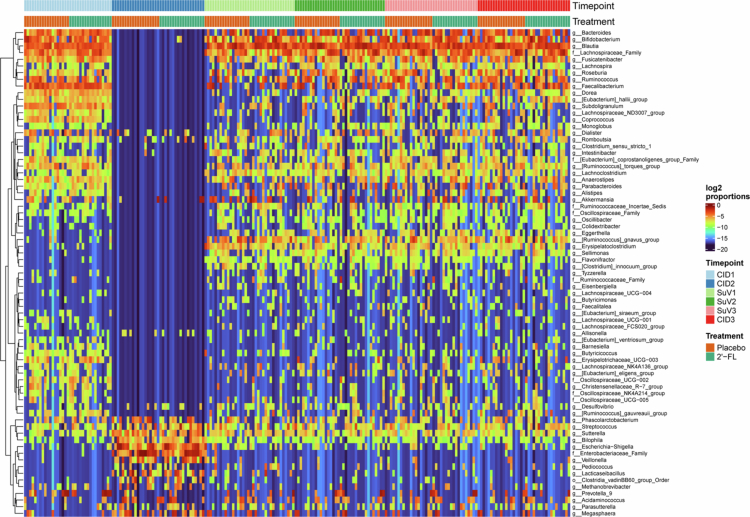
Heatmap of gut microbial composition based on log2-transformed genus-level taxonomic abundance throughout the study period, coloured by timepoint and treatment. 2′-FL: 2′-fucosyllactose; CID: Clinical Investigation Day; SuV: Supplementary Visit. CID2, CID3, SuV1, SuV2 and SuV3 2′-FL: *n* = 18; CID1 2′-FL: *n* = 17; CID2, CID3, and SuV3 Placebo: *n* = 19; CID1, SuV1 and SuV2 Placebo: *n* = 18.

Vancomycin use markedly disrupted gut microbial composition: 58/73 bacterial taxa changed significantly (*q* < 0.05) (Figure 3). Among these taxa, the relative abundance of 36 taxa decreased during vancomycin use, including the genera *Bifidobacterium*, *Faecalibacterium*, *Blautia* and *Roseburia* (Suppl. Table 3). For the other 22 taxa, including the family *Enterobacteriaceae* and the genera *Streptococcus, Veillonnella* and *Lacticaseibacillus,*relative abundance increased significantly upon vancomycin use.

### 2′-FL transiently improves gut microbial resilience after antibiotic use

**Figure 4.**
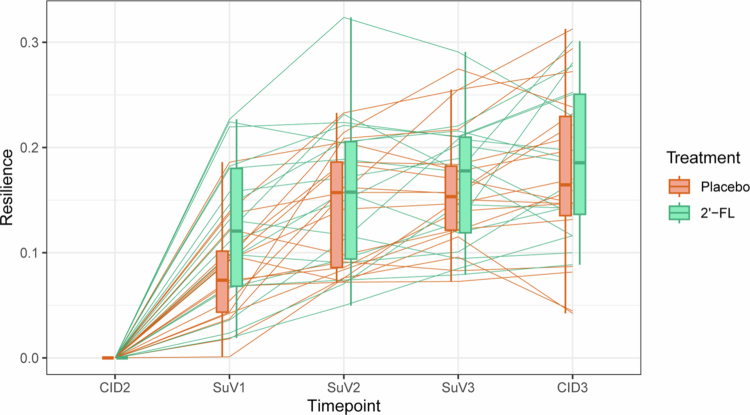
Gut microbial resilience after seven-day vancomycin use and throughout eight-week 2′-FL or placebo supplementation. 2′-FL: 2′-fucosyllactose, CID: Clinical Investigation Day, SuV: Supplementary Visit. CID2, SuV2, SuV3 and CID3 Placebo: *n* = 18; SuV1 Placebo: *n* = 17; CID2, SuV1, SuV2, SuV3 and CID3 2′-FL: *n* = 18.

To investigate the ability of the gut microbiome to recover from vancomycin-induced perturbations, microbial resilience was calculated. The resilience was lowest directly after antibiotic use, the moment of maximum perturbation (Figure 4). Eight-week 2′-FL supplementation did not improve gut microbial resilience compared to placebo (*p*Treatment*Time = 0.832). However, a differential change in resilience over time was observed during the first two weeks after vancomycin use (*p*Treatment*Time = 0.043), as there was a large increase in the 2′-FL group compared to placebo (Figure 4). Additionally, after two weeks of supplementation (SuV1), resilience was significantly higher in the 2′-FL group compared to placebo (0.12 + /−0.02 vs 0.08 + /−0.01; *p* = 0.044). Together, this indicates a beneficial, yet temporary, effect of 2′-FL supplementation on microbiota resilience upon vancomycin-induced perturbations.

### No improved recovery of microbial diversity with 2′-FL supplementation

Eight-week 2′-FL supplementation did not significantly improve the recovery of gut microbial richness and diversity (*p*Treatment*Time = 0.611 & *p*Treatment*Time = 0.418) (Figure 2A-B). Notably, both observed genus richness and Shannon diversity were still significantly lower after eight-week 2′-FL supplementation compared to baseline (*p* = 0.005 & *p* = 0.030, respectively). The observed genus richness also remained decreased after eight-week placebo supplementation as compared to baseline (*p* = 0.005), while the Shannon diversity did not (*p* = 0.167). We further investigated changes in microbial richness and diversity during the first two weeks of supplementation to gain insight into this period of recovery. Even though resilience increased, there was no differential change in observed genus richness and Shannon diversity during the first two weeks of 2′-FL supplementation compared to placebo (*p* = 0.807 & *p* = 0.471, respectively) (Figure 2A-B). This indicates that while recovery of richness and diversity did not differ between groups, there was a larger recovery in the overall gut microbial community structure towards baseline during two-week 2′-FL supplementation compared to placebo.

In order to assess the impact of baseline gut microbial richness and diversity on microbial resilience, both the placebo and the 2′-FL group were split based on the median baseline observed genus richness as well as Shannon diversity. Gut microbial resilience was significantly higher after four weeks of 2′-FL supplementation in individuals with a lower baseline Shannon diversity compared to those with higher diversity (0.19 + /−0.08 vs. 0.13 + /−0.05; *p* = 0.045) (Suppl. Figure 1).

### 2′-FL supplementation impacts the recovery of specific bacterial taxa

Over the eight-week supplementation period, the genera *Parabacteroides* (*p*Treatment*Time = 0.040) and *Eisenbergiella* (*p*Treatment*Time = 0.017) changed differentially between the 2′-FL and placebo group (Suppl. Table 4), a finding which may imply effects of 2′-FL on specific taxa. During the first two weeks of supplementation, several taxa were affected differently, and changes were clearer than the trends over eight weeks: the relative abundance of *Escherichia–Shigella* decreased more in the 2′-FL group compared to placebo (*p*Treatment*Time = 0.017) (Suppl. Table 4). *Streptococcus* decreased in the 2′-FL group, while it expanded in the placebo group (*p*Treatment*Time = 0.032). The relative abundance of the *[Eubacterium] hallii group* increased with two-week 2′-FL supplementation, while it decreased in the placebo group (*p*Treatment*Time = 0.027), and *Roseburia* expanded more in the placebo group (*p*Treatment*Time = 0.037). These findings support the role of a transient effect of 2′-FL supplementation after seven-day vancomycin use.

### Gut microbial resilience associates with specific bacterial taxa

To further investigate whether gut microbial resilience was associated with differences in the gut microbiome, we correlated resilience after two weeks of 2′-FL or placebo supplementation with microbial *α* diversity and bacterial taxa. There were no significant correlations between microbial resilience and measures of *α* diversity after two weeks of 2′-FL supplementation (Suppl. Table 5). Notably, relative abundance of specific taxa correlated with resilience after two weeks of 2′-FL intake but not placebo: *Bacteroides* (*ρ* = 0.605, *p* = 0.010), *Megasphaera* (*ρ* = -0.583, *p* = 0.014), *Lacticaseibacillus* (*ρ* = −0.569, *p* = 0.017), *Parabacteroides* (*ρ* = 0.517, *p* = 0.034), *Parasutterella* (*ρ* = 0.490, *p* = 0.046), *[Eubacterium] hallii group* (*ρ* = -0.517, *p* = 0.034), *Alistipes* (*ρ* = 0.529, *p* = 0.029), *Lachnospiraceae NK4A136 group* (*ρ* = −0.637, *p* = 0.006) and the order *Clostridia vadinBB60 group* (*ρ* = −0.485, *p* = 0.048) (Suppl. Table 5). However, after correction for multiple testing, these correlations were no longer significant.

### 2′-FL supplementation does not improve recovery of faecal SCFA concentrations

Aside from microbial composition, gut microbial function may be more relevant in determining the consequences of antibiotic use on host health. Our analyses show reduced fecal concentrations of the SCFAs acetate, butyrate and propionate after vancomycin use (all *p* < 0.001) (Table 2). Concentrations of fecal valerate, caproate and the branched-chain fatty acids (BCFAs) isobutyrate and isovalerate also decreased significantly (all *p* < 0.001). Conversely, the fasting plasma concentration of the gut hormone GLP−1 increased during vancomycin use (10.5 + /−0.71 pmol/L vs. 12.0 + /−0.65; *p* = 0.009), whilst no changes in GLP−1 concentrations were observed after the oral glucose load. Eight-week 2′-FL supplementation did not have a differential impact on the recovery of fecal SCFA and BCFA concentrations compared to placebo (Table 2). There were no differential changes in fasting and 2-h plasma GLP−1 concentrations between 2′-FL and placebo supplementation (*p*Treatment*Time = 0.199 and 0.313).

**Table 2.** Faecal SCFA and BCFA, and plasma GLP−1 concentrations throughout the study period.

	Total group			Baseline		Post vancomycin		Follow-up		*p*Treatment *Time
	Baseline	Post vancomycin	*p*-value	Placebo	2′-FL	Placebo	2′-FL	Placebo	2′-FL	
Acetate (mmol/L)	56.24 + /−3.14	26.58 + /−1.14	<0.001	59.71 + /−3.57	52.57 + /−5.21	28.08 + /−1.47	25.40 + /−1.68	48.82 + /−2.76	46.18 + /−3.27	0.993
Butyrate (mmol/L)	16.63 + /−1.44	6.30 + /−0.33	<0.001	17.72 + /−2.07	15.47 + /−2.02	6.39 + /−0.47	5.98 + /−0.49	13.50 + /−1.21	13.29 + /−1.60	0.920
Propionate (mmol/L)	18.41 + /−1.45	9.53 + /−0.52	<0.001	18.20 + /−1.74	18.64 + /−2.41	9.82 + /−0.63	9.31 + /−0.82	15.46 + /−1.24	12.93 + /−1.02	0.216
										
Valerate (mmol/L)	1.50 + /−0.28	0.28 + /−0.09	<0.001	1.32 + /−0.16	1.69 + /−0.55	0.35 + /−0.16	0.31 + /−0.14	0.97 + /−0.19	0.75 + /−0.11	0.490
Caproate (mmol/L)	0.48 + /−0.10	0.12 + /−0.02	<0.001	0.52 + /−0.15	0.44 + /−0.14	0.18 + /−0.07	0.12 + /−0.03	0.20 + /−0.05	0.19 + /−0.04	0.665
Iso-butyrate (mmol/L)	0.87 + /−0.08	0.22 + /−0.02	<0.001	0.77 + /−0.09	0.99 + /−0.13	0.30 + /−0.09	0.23 + /−0.03	0.62 + /−0.07	0.58 + /−0.07	0.838
Iso-valerate (mmol/L)	0.68 + /−0.07	0.22 + /−0.03	<0.001	0.56 + /−0.07	0.80 + /−0.12	0.28 + /−0.08	0.25 + /−0.04	0.49 + /−0.06	0.43 + /−0.05	0.824
										
Fasting GLP−1 (pmol/L)	10.5 + /−0.71	12.0 + /−0.65	0.009	10.7 + /−1.12	10.4 + /−0.91	11.9 + /−0.87	12.1 + /−0.99	11.6 + /−0.95	10.0 + /−1.04	0.199
2-hour GLP−1 (pmol/L)	17.2 + /−1.18	17.2 + /−1.19	0.822	17.4 + /−1.72	16.9 + /−1.66	17.1 + /−1.63	17.3 + /−1.78	18.9 + /−3.15	16.1 + /−2.18	0.313

2′-FL: 2′-fucosyllactose; GLP−1: glucagon-like peptide 1. Total group: *n* = 37; Placebo: *n* = 19; 2′-FL: *n* = 18.

### No effect of 2′-FL supplementation on parameters of metabolic health

To investigate the impact of seven-day vancomycin use and subsequent 2′-FL supplementation on host metabolic health, we performed extensive metabolic phenotyping at three timepoints throughout the study period. Seven-day vancomycin use did not affect measures of whole-body insulin sensitivity, such as HOMA-IR and the Matsuda index, or tissue-specific insulin sensitivity, as assessed by HIRI, MISI and ADIPO-IR, nor did it affect the insulinogenic index, a surrogate marker for insulin secretion (Table 3). Notably, fasting plasma total cholesterol decreased significantly during vancomycin use (4.92 + /−0.17 mmol/L vs. 4.79 + /−0.16; *p* = 0.017), and the concomitant decrease in fasting LDL tended to be significant (*p* = 0.055). Eight-week supplementation did not lead to differential changes in markers of glucose metabolism and insulin sensitivity. Fasting plasma total cholesterol increased during the supplementation period, with a tendency toward a smaller increase in the 2′-FL group compared to placebo (*p*Treatment*Time = 0.085).

**Table 3.** Parameters of metabolic health throughout the study period.

	Total group			Baseline		Post vancomycin		Follow-up		*p*Treatment *Time
	Baseline	Post vancomycin	*p*-value	Placebo	2′-FL	Placebo	2′-FL	Placebo	2′-FL	
Weight (kg)	89.8 + /−2.9	89.6 + /−2.9	0.285	87.2 + /−3.9	92.4 + /−4.2	87.2 + /−3.9	92.1 + /−4.2	87.3 + /−4.0	91.9 + /−4.3	0.607
BMI (kg/m^2)	30.4 + /−0.7	30.4 + /−0.7	0.248	29.5 + /−0.9	31.4 + /−1.0	29.5 + /−0.9	31.3 + /−1.0	29.5 + /−0.9	31.2 + /−1.0	0.747
										
Waist (cm)	98.5 + /−2	98.2 + /− 1.9	0.599	94.4 + /−2.4	102.5 + /−3.0	94.6 + /−2.2	101.9 + /−3.0	94.2 + /−2.4	101.0 + /−3.4	0.716
Hip (cm)	106.3 + /−1.5	105.9 + /−1.5	0.708	103.3 + /−1.7	109.3 + /−2.4	103.6 + /−1.5	108.2 + /−2.5	103.2 + /−1.8	106.8 + /−2.6	0.504
W/H ratio	0.92 + /−0.01	0.93 + /−0.01	0.698	0.91 + /−0.01	0.94 + /−0.01	0.91 + /−0.02	0.94 + /−0.01	0.91 + /−0.01	0.094 + /−0.02	0.965
										
HOMA-IR	1.7 + /−0.17	1.7 + /−0.16	0.905	1.6 + /−0.17	1.9 + /−0.30	1.5 + /−0.18	1.8 + /−0.27	1.7 + /−0.22	1.9 + /−0.29	0.400
Matsuda Index	18.7 + /−2.6	18.5 + /−2.0	0.388	16.5 + /−2.0	18.6 + /−4.4	18.3 + /−2.0	16.9 + /−3.1	17.5 + /v 1.8	15.6 + /− 2.6	0.806
HIRI	491 + /−43	436 + /−36	0.106	452 + /−45	531 + /−76	413 + /−44	460 + /−58	360 + /−58	534 + /−75	0.156
MISI	0.179 + /−0.024	0.166 + /−0.022	0.713	0.155 + /− 0.023	0.205 + /−0.043	0.193 + /− 0.035	0.138 + /−0.024	0.221 + /− 0.042	0.138 + /−0.032	0.852
ADIPO-IR	28.4 + /−2.9	28.0 + /− 3.1	0.729	27.4 + /−3.2	29.5 + /−4.9	24.8 + /−3.5	31.3 + /− 5.1	28.5 + /−4.3	29.3 + /−4.8	0.196
HOMA-β	120 + /− 14	112 + /−11	0.617	120 + /−21	119 + /− 17	105 + /−15	119 + /−16	118 + /−18	118 + /−15	0.226
Insulinogenic Index	44.9 + /−3.7	40.7 + /− 2.8	0.138	41.4 + /−3.9	48.5 + /− 6.4	38.6 + /−3.7	42.9 + /− 4.3	40.1 + /−7.2	46.2 + /−5.6	0.755
Disposition Index	619 + /−43	603 + /−35	0.546	605 + /−53	634 + /− 70	623 + /−46	582 + /− 55	572 + /−66	561 + /−55	0.711
										
Fasting Glucose (mmol/L)	4.92 + /−0.08	4.93 + /− 0.06	0.753	4.87 + /−0.10	4.98 + /−0.13	4.92 + /−0.08	4.94 + /− 0.11	4.93 + /−0.08	5.01 + /−0.13	0.438
Fasting Insulin (pmol/L)	54.8 + /−5.14	56.0 + /−5.23	0.869	50.7 + /−5.29	59.1 + /−9.03	48.9 + /−5.83	57.6 + /− 8.19	54.5 + /−6.85	57.7 + /−8.15	0.313
2-hour Glucose (mmol/L)	5.26 + /-0.23	5.38 + /−0.23	0.424	4.87 + /−0.36	5.68 + /− 0.27	5.15 + /−0.32	5.62 + /− 0.33	5.35 + /−0.35	5.62 + /−0.39	0.570
										
Free Fatty Acids (umol/L)	533 + /−28	535 + /−35	0.790	553 + /−46	511 + /−32	524 + /−62	548 + /−33	532 + /−50	517 + /−39	0.504
Triglycerides (mmol/L)	1.44 + /−0.18	1.28 + /−0.12	0.083	1.27 + /−0.12	1.61 + /−0.35	1.17 + /−0.12	1.40 + /− 0.21	1.29 + /−0.10	1.43 + /−0.22	0.388
Total Cholesterol (mmol/L)	4.92 + /−0.17	4.79 + /−0.16	0.017	4.78 + /−0.16	5.07 + /−0.30	4.60 + /−0.17	4.99 + /−0.27	5.02 + /−0.19	5.15 + /−0.23	0.085
HDL (mmol/L)	1.26 + /−0.05	1.27 + /−0.05	0.455	1.26 + /−0.07	1.27 + /−0.07	1.27 + /−0.08	1.28 + /−0.07	1.26 + /−0.07	1.25 + /−0.07	0.734
LDL (mmol/L)	3.01 + /−0.11	2.93 + /−0.12	0.055	2.95 + /−0.15	3.08 + /−0.18	2.80 + /−0.15	3.08 + /−0.19	3.18 + /−0.17	3.25 + /−0.19	0.190

2′-FL: 2'-fucosyllactose; BMI: body mass index; W/H ratio: waist-to-hip ratio; HOMA-IR: homeostatic model assessment of insulin resistance; HIRI: hepatic insulin resistance index; MISI: muscle insulin sensitivity index; ADIPO-IR: adipose tissue insulin resistance index; HOMA-β: homeostatic model assessment of β-cell function; HDL: high-density lipoprotein; LDL: low-density lipoprotein. Total group: n=37; placebo group: n=19; 2′-FL group: n=18.

Fasting plasma inflammatory cytokines did not change under seven-day vancomycin use (Table 4). Interestingly, IL−6 decreased during eight-week 2′-FL supplementation (0.84 + /−0.07 pg/ml vs. 0.75 + /−0.05) while it increased in the placebo group (0.88 + /−0.12 vs. 0.97 + /−0.13) (*p*Treatment*Time = 0.041) (Table 4).

**Table 4.** Plasma inflammatory cytokines throughout the study period.

	Total group			Baseline		Post vancomycin		Follow-up		*p*Treatment *Time
	Baseline	Post vancomycin	*p*-value	Placebo	2′-FL	Placebo	2′-FL	Placebo	2′-FL	
IL−6 (pg/ml)	0.83 + /−0.09	0.86 + /−0.07	0.183	0.93 + /−0.15	0.72 + /−0.06	0.88 + /−0.12	0.84 + /−0.07	0.97 + /−0.13	0.75 + /−0.05	0.041
IL−8 (pg/ml)	5.75 + /−0.30	5.66 + /−0.28	0.802	5.69 + /−0.33	5.81 + /−0.52	5.93 + /−0.42	5.39 + /−0.36	5.78 + /−0.33	5.51 + /−0.47	0.575
IL−10 (pg/ml)	0.28 + /−0.04	0.28 + /−0.02	0.904	0.32 + /−0.07	0.25 + /−0.02	0.27 + −0.03	0.29 + /−0.02	0.26 + /−0.03	0.28 + /−0.03	0.629
TNF-*α* (pg/ml)	1.35 + /−0.05	1.38 + /−0.04	0.228	1.38 + /−0.07	1.32 + /−0.06	1.39 + /−0.05	1.38 + /−0.06	1.43 + /−0.08	1.39 + /−0.09	0.738
IFN-*γ* (pg/ml)	7.64 + /−2.20	5.53 + /−0.50	0.335	9.16 + /−4.27	6.03 + /−0.68	4.48 + /−0.24	6.58 + /−0.91	5.43 + /−0.59	6.83 + /−1.10	0.589

2′-FL: 2′-fucosyllactose; IL: Interleukin; TNF-α: Tumor Necrosis Factor α; IFN-γ: Interferon-γ. Total group: n=37; placebo: n=19; 2′-FL: n=18.

### Gut microbial resilience does not associate with parameters of metabolic health

Finally, we correlated gut microbial resilience after two weeks of 2′-FL or placebo supplementation with markers of metabolic health. Resilience after two weeks of placebo supplementation was positively correlated with body weight after eight weeks (*ρ* = 0.603, *p* = 0.010) (Suppl. Table 6). Resilience after two weeks of 2′-FL supplementation correlated positively with waist circumference (*ρ* = 0.603, *p* = 0.010), disposition index (*ρ* = 0.517, *p* = 0.034) faecal acetate concentrations (*ρ* = 0.490, *p* = 0.046), and faecal caproate (*ρ* = 0.529, *p* = 0.029), while it negatively correlated with 2-h glucose (*ρ* = −0.583, *p* = 0.014), MISI (*ρ* = −0.569, *p* = 0.017) and faecal propionate (*ρ* = −0.517, *p* = 0.034). Again, with correction for multiple testing, these correlations were no longer significant.

## Discussion

In this double-blind placebo-controlled, randomized human intervention study, we showed that 2′-FL supplementation following a seven-day course of oral vancomycin significantly improved short-term (two-week) gut microbial resilience compared to placebo in adults with overweight or obesity. However, no differences in microbial resilience were observed in the long term (eight weeks).

Overall, seven-day oral vancomycin use clearly disrupted gut microbial diversity and composition and reduced fecal SCFA concentrations, while limited changes were found in parameters of metabolic health. There was a larger increase in gut microbial resilience after the first two weeks of 2′-FL supplementation compared to placebo, indicating that 2′-FL improved initial microbial recovery after vancomycin use. Eight-week 2′-FL intake resulted in reduced plasma IL-6 concentrations compared to placebo.

### Vancomycin use results in perturbations of the gut microbiome

The disruptive effect of vancomycin on the gut microbiome is well established. Consistent with previous findings, our study shows that a seven-day course of oral vancomycin in adults with overweight or obesity significantly reduces microbial richness and diversity, aligning with reports of similar declines following both seven- and 14-d vancomycin regimens.^14-16^ Further, in line with our results, previous studies among individuals with normal weight, overweight, obesity and metabolic syndrome^14-16^^,^^43^ have consistently reported a depletion in various bacterial taxa upon oral vancomycin exposure, including a decrease in *Faecalibacterium prausnitzii, Anaerobutyricum* (formerly *Eubacterium*) *hallii, Roseburia* (*hominis*), *Coprococcus, Ruminococcus, Dorea, Blautia* and *Anaerostipes hadrus*. Furthermore, these studies generally showed an increased relative abundance of *Escherichia coli, Klebsiella, Enterobacter, Lactobacillus* (recently split into *Lactiplantibacillus* and *Lacticaseibacillus,* among others) and *Veillonella* after vancomycin treatment, in accordance with our results. *E. coli* and *Klebsiella (pneumoniae)* are considered opportunistic pathogens that can bloom as a result of the depletion of other members of the gut microbiota, potentially leading to complications.^44^^,^^45^ Interestingly, the clear reduction in bifidobacteria in our study is not commonly reported, even though these bacteria are susceptible to vancomycin. Only one previous study reported a decrease in *Bifidobacterium* after seven-day oral vancomycin use in adult males (BMI: 18−28 kg/m^2^).^43^

### 2′-FL supplementation improves gut microbial resilience

Our results showed a differential change in gut microbial resilience, defined as the ability of the gut microbiome to return to its baseline state after perturbation, during the first two weeks of 2′-FL supplementation after vancomycin use compared to placebo, with a larger increase in resilience in the group receiving 2'-FL. Over a longer period however, 2′-FL did not improve the recovery of the gut microbiome compared to placebo. Notably, the gut microbiome had not fully recovered back to baseline after eight weeks of either 2′-FL or placebo supplementation, as richness was still lower compared to baseline, and the gut community structure had shifted. This is in agreement with the literature showing an incomplete recovery of the gut microbiome after vancomycin use.^15^ Many bifidobacteria are known to efficiently utilize 2′-FL as a carbon source,^23^ generating intermediary metabolites that can support bacterial cross-feeding and promote the growth of other taxa, including butyrate producers.^46^ However, the disruptions caused by vancomycin are substantial and may exceed what can be restored through 2′-FL supplementation alone. A recent study did show the potential of 2′-FL to improve recovery in an *in vitro* model of the colon, as the gut microbiota from one-month old infants supplemented with 2′-FL recovered faster after disruption by amoxicillin/clavulanate compared to galacto-oligosaccharide supplementation or control.^47^

Concurrent with the observed improvements in gut microbial resilience after two weeks of supplementation, the relative abundance of *Escherichia-Shigella* and *Streptococcus* decreased during the two-week 2′-FL supplementation period compared to placebo. These bacterial taxa include many opportunistic pathogens, and these have expanded under vancomycin use. Supplementation of 2′-FL seems to have had a beneficial impact by more extensively promoting the expansion of common intestinal bacteria that outcompete these potential pathogens compared to placebo, thereby leading to a decrease in their relative abundance. It is well known that bacteria employ several mechanisms to inhibit pathogen growth in the gut, so-called colonization resistance.^48^ Additionally, the relative abundance of *the [Eubacterium] hallii group* increased during the first two weeks of 2′-FL supplementation, while it decreased with placebo. *Eubacterium hallii* (now *Anaerobutryicum*) has been shown to be a butyrate and propionate producer and is able to use 1,2-propanediol (1,2-PD), produced from L-fucose, which in turn is a metabolite resulting from 2′-FL breakdown.^49^^,^^50^ Finally, the relative abundance of *Roseburia* increased more during the first two weeks of placebo supplementation compared to 2′-FL. The genus *Roseburia* includes several well-known SCFA-producing species, and it may have beneficial effects for gut and host health.^51^

While 2′-FL and its metabolites may be utilized by bacteria in order to expand, 2′-FL can also impact the gut microbiome and host through direct signaling effects. Previous work has highlighted that the structural-functional properties of 2′-FL elicit responses in the gut epithelium, via which inflammation may be modified, which may subsequently lead to changes in the gut microbiota.^52^

Although 2′-FL supplementation stimulated microbial resilience in the short term compared to placebo, there was no difference over a period of eight weeks. The limited impact of 2′-FL supplementation over time was further evidenced by the fact that only two taxa changed differentially under eight-week 2′-FL supplementation compared to placebo: *Parabacteroides* and *Eisenbergiella*. In a cohort of adults with obesity, the relative abundance of *Parabacteroides* was negatively associated with body weight, blood glucose and serum lipids.^53^ A lower relative abundance of *Eisenbergiella* has been found in individuals with nonalcoholic fatty liver disease (NAFLD) compared to healthy controls.^54^ Our data indicate that 2′-FL may have been too limited to promote well-balanced recovery over a longer period. A mix of prebiotic compounds targeting a wider range of bacteria could have had more beneficial effects for the recovery of microbial richness, diversity and composition, steering the gut microbial community more completely towards baseline. Future research should further investigate the concept of gut microbial resilience, including factors that influence it and strategies to increase this resilience. A key factor to consider is the baseline gut microbiome, an important determinant of the response to intervention.^18^ Results from our work may indicate that individuals with a microbiota with a lower richness and diversity could benefit more from 2′-FL supplementation after disruption (Suppl. Figure 1). Future research is needed, however, as our study was not sufficiently powered to analyze this, and only cross-sectional comparisons were performed.

### Faecal SCFA concentrations were affected by vancomycin use but not by 2′-FL

Seven-day vancomycin use significantly decreased fecal SCFA and BCFA concentrations, a finding in agreement with previous studies showing either decreased fecal or plasma SCFAs after vancomycin use.^15^^,^^16^ This change can be explained by the disruptive effect of vancomycin on the presence of SCFA producers in the gut, for example, the Gram-positive bacteria belonging to the *Lachnospiraceae* and *Oscillospiraceae* families^55^ Our results further show no changes in postprandial plasma GLP-1 after vancomycin use, which is consistent with previous studies.^14-16^ In contrast, our results do show a slight but significant increase in fasting GLP−1 concentrations, of which the clinical significance remains to be determined.

Eight-week 2′-FL supplementation did not improve the recovery of fecal SCFA concentrations compared to placebo. Additionally, there was no differential change in faecal BCFA concentrations in this period. Previous studies have shown the ability of 2′-FL to stimulate SCFA production: *in vitro*, fermentation of 2 g/L 2′-FL by fecal microbiota from formula-fed infants resulted in increased SCFA production, mainly butyrate, and a bifidogenic effect.^56^ More importantly, previous work from our group showed that one-day intake of 2′-FL (12 g) led to increased postprandial plasma acetate and butyrate concentrations in lean males and postprandial butyrate in males with overweight or obesity, indicating the potential of 2′-FL to acutely stimulate SCFA production.^27^ Our results indicate that eight-week supplementation of 2′-FL was insufficient to elicit a different response in recovery compared to placebo. We cannot exclude that there may have been beneficial effects of 2′-FL supplementation for fecal SCFA concentrations in the short term (two weeks of supplementation), as we did not analyze SCFAs at that timepoint. Future work should therefore include more detailed analyses at timepoints during the early stages of recovery, as changes in this critical window may have more extensive impact.

### 2′-FL did not affect insulin sensitivity but decreased plasma IL-6

Seven-day vancomycin use did not lead to changes in indices of whole-body and tissue-specific insulin sensitivity, a finding in accordance with studies in literature.^15^^,^^16^ A decreased peripheral insulin sensitivity was reported after seven-day oral vancomycin use in males with obesity and metabolic syndrome compared to baseline, but this study did not include a control group.^14^

Eight-week 2′-FL supplementation did not differentially impact markers of metabolic health, but did lead to a differential effect on fasting plasma IL−6: concentrations decreased with 2′-FL supplementation but increased in the placebo group, indicating potential anti-inflammatory effects of 2'-FL. A murine study investigating a model of intestinal colitis previously showed that fermentation of 2'-FL by the gut microbiota minimised intestinal inflammation, a process suggested to be influenced by expansion of *Ruminococcus gnavus* and increased propionate production.^57^ Additionally, previous work has implied that SCFAs resulting from saccharolytic fermentation, mainly butyrate, could be mediators of an anti-inflammatory effect, besides other benefits.^58^ Moreover, 2′-FL may have anti-inflammatory effects through its signalling function in the intestinal epithelium, as it has been shown to be able to directly interact with immune cells.^59^ In our study, eight-week supplementation with 2′-FL may have had beneficial anti-inflammatory effects, although we did not see differences in faecal SCFA concentrations. Finally, microbial resilience after two weeks of 2′-FL supplementation did not correlate with parameters of metabolic health after eight weeks.

## Conclusion and outlook

To the best of our knowledge, this is the first study investigating the potential of prebiotic supplementation to improve gut microbial resilience after antibiotic use in humans. This unique aspect of our study underlines the importance of considering resilience in human studies on antibiotic-induced dysbiosis. Other strengths of this study are the in-depth phenotyping of our participants as well as the detailed investigation of antibiotic-induced gut microbial disruption and subsequent recovery, allowing us to extensively describe the impact of vancomycin use and 2′-FL supplementation. One limitation of this work is that the changes in the gut microbiota resulting from vancomycin use and 2′-FL supplementation are only investigated on a compositional level. Further investigation into gut microbial functionality using metagenomic sequencing will shed further light on the process of prebiotic-stimulated recovery of the gut microbiome after antibiotic use by analysing microbial functional genes, potentially highlighting key functions of a more resilient gut microbiome. Still, we feel that our work on taxonomic resilience, consistent with ecological theory, offers important insights into the most important factor of microbial recovery, a stable structural configuration. From this, one can expand their investigation into the functional potential of the microbiota.

Another limitation of our work was the limited study population, as the study was performed in a single center, and volunteers came from the area around Maastricht, The Netherlands. As the microbiome differs widely between individuals, more research is needed to assess whether our results would be applicable to a wider population. Similarly, the gut microbiome is impacted by an individual’s sex. A limitation of our work was that it was not powered to compare effects in males versus females.

In conclusion, 2′-FL supplementation led to initial transient improvements in gut microbial resilience after vancomycin use, indicating its beneficial potential. There were no major effects on host metabolic health, only a tendency towards reduced plasma IL-6. Of note, eight-week 2′-FL supplementation did not lead to a full recovery of the microbiome after antibiotic use, as the community structure was still shifted compared to baseline. Whether the results found in this study can be translated to other antibiotics and prebiotics remains to be studied. At any rate, this study gives insights into how prebiotic supplementation may have beneficial effects on microbial recovery in the short term by stimulating resilience. Further research is needed to elucidate the drivers of gut microbial resilience and increase the effectiveness of supplementation strategies by defining the best prebiotic compound(s). As antibiotic courses are often urgently prescribed, a supplementation strategy after antibiotic use would be beneficial to limit disruption, especially for at-risk populations.

## Supplementary Material

Supplementary Material Supplementary Table 2: Dietary intake and physical activity score at baseline and follow-up.

Supplementary Material Supplementary

Supplementary Material Supplementary Material

## Data Availability

The sequencing data for this study have been deposited in the European Nucleotide Archive (ENA) at EMBL-EBI under accession number PRJEB80391 (https://www.ebi.ac.uk/ena/browser/view/PRJEB80391). Other data can be made available by the corresponding author upon reasonable request.
