# Supplementary figures and images for: 2′-Fucosyllactose supplementation results in a transient improvement in gut microbial resilience after vancomycin use in adults with overweight or obesity: a randomized, double-blind, placebo-controlled intervention
Source: Gut Microbes. 2025 Nov 16;17(1):2580693. doi: 10.1080/19490976.2025.2580693 (PMC12629329; doi:10.1080/19490976.2025.2580693)

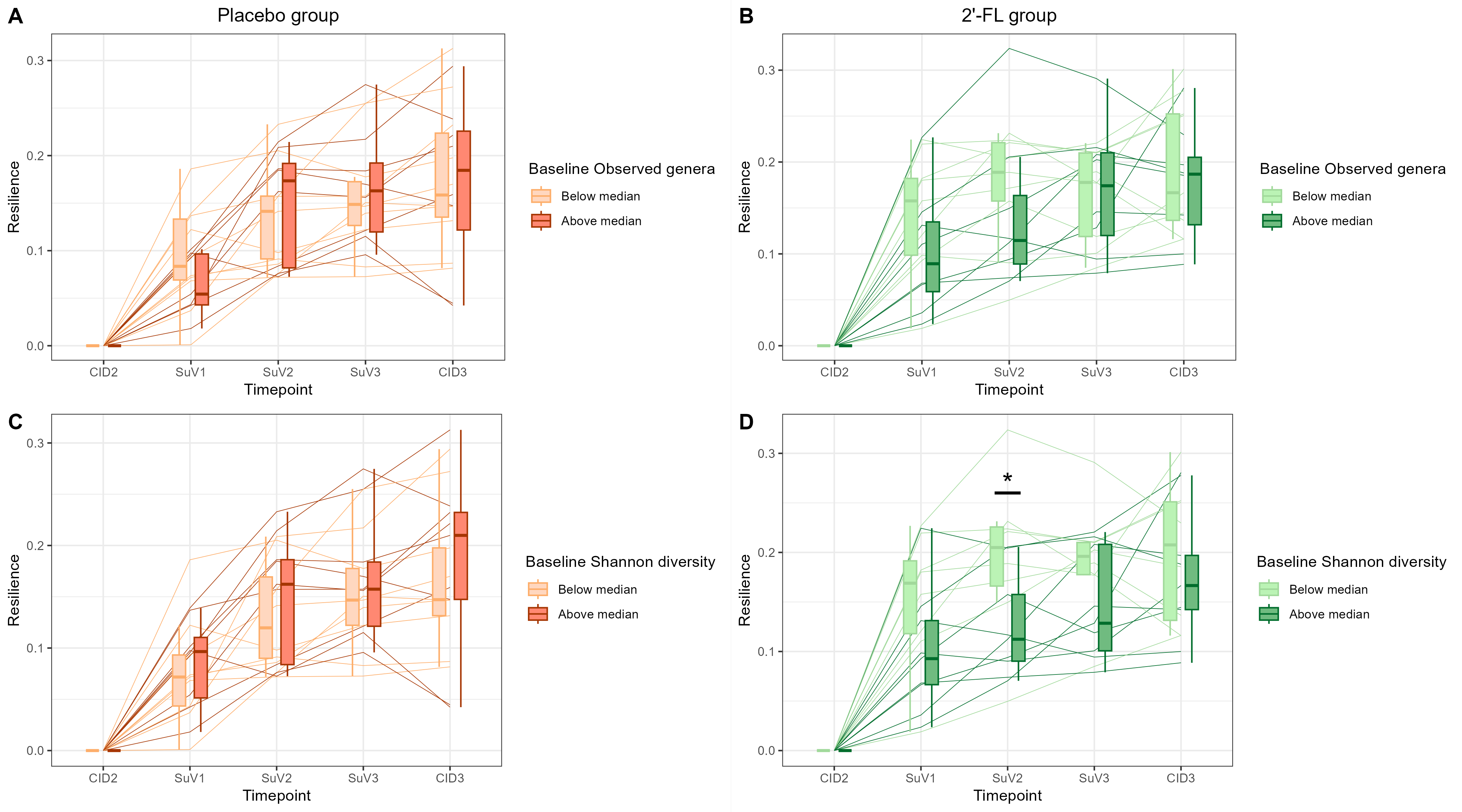

Supplement: Supplementary Material — Supplementary [file KGMI_A_2580693_SM4925.tiff]
