# Supplementary material for: 2′-Fucosyllactose supplementation results in a transient improvement in gut microbial resilience after vancomycin use in adults with overweight or obesity: a randomized, double-blind, placebo-controlled intervention
Source: Gut Microbes. 2025 Nov 16;17(1):2580693. doi: 10.1080/19490976.2025.2580693 (PMC12629329; doi:10.1080/19490976.2025.2580693)
Supplement: Supplementary Material [file KGMI_A_2580693_SM3767.docx]

Supplementary Figure

Supplementary Figure 1: Gut microbial resilience over time in the placebo group split based on median baseline observed genera (A) and median baseline Shannon diversity (C), and in the 2’-FL group split based on median baseline observed genera (B) and median baseline Shannon diversity (D).

Supplementary Tables

Supplementary Table 1A: Bristol Stool Chart score throughout the study period.

Supplementary Table 1B: Gastrointestinal Symptom Rating Scale scores at baseline and post vancomycin.

Supplementary Table 1C: Gastrointestinal Symptom Rating Scale scores throughout the study period.

Supplementary Table 2: Dietary intake and physical activity score at baseline and follow-up

Supplementary Table 3: Relative abundance of filtered taxa, comparing baseline and post vancomycin.

Supplementary Table 4: Bacterial taxa with a differential change under eight-week or two-week 2'-FL supplementation compared to placebo.

Supplementary Table 5: Correlations between microbial resilience and α-diversity or bacterial taxa after two weeks of 2'-FL or placebo supplementation.

Supplementary Table 6: Correlations between microbial resilience after two weeks of 2'-FL or placebo supplementation and parameters of metabolic health after eight weeks.
